# Optimization of *Pseudomonas aeruginosa* isolated for bioremediation from Ha'il region of Saudi Arabia

**DOI:** 10.6026/97320630019893

**Published:** 2023-09-30

**Authors:** K. D Alsukaibi Abdulmohsen, Fathi Rabeh Alimi, Lassaad Mechi, Otaibi Ahmed A., Alshamari Asma K.A.A, Alshammari Eida Mohammad, Mohd Wajid Ali Khan

**Affiliations:** 1 Department of Chemistry, College of Sciences, University of Ha'il, Ha'il 55473, Saudi Arabia; 2 Medical and Diagnostic Research Center, University of Ha'il, Ha'il, 55473, Saudi Arabia

**Keywords:** Methyl red dyes, Wastewater, *Pseudomonas aeruginosa*, Bioremediation, Decolorization

## Abstract

Majority of dyes are toxic to all the living organisms and inherently resistant to microbial degradation. Hence, decolorization and degradation of textile dye methyl red were evaluated using isolated bacterial strain *Pseudomonas aeruginosa* (*P. aeruginosa*). Methyl red dye decolorization by *P. aeruginosa* with respect to various parameters was optimized. Data shows that maximum possible decolorization was seen at 50 ppm dye concentration, 1400 mg/l glucose concentration, 700 mg/l sodium chloride (NaCl) concentration, pH 9, temperature 38°C, 1000 mg/l urea concentration *P. aeruginosa* AM-1 strain. The highest percent (91.1%) of bioremediation was achieved at 40 ppm dye concentration in *Allium cepa* test. These findings suggest *P. aeruginosa* strain (AM-1) has the potential to be used in the biological treatment of highly toxic dye which is main constituent of dyeing mill effluents due to its high decolorization activity with simple conditions. Strain AW-1 strain also has potential to bioremediate other wastewater containing methyl red dye.

## Background:

Dye contaminants are found in lakes, rivers, and wastewater, because dyes limit sunlight, inhibits photosynthesis, and reduces oxygen mass transfer [1]. The textile sector is the biggest user of dyes, and synthetic dyestuffs are widely used in a variety of business sectors, including food, cosmetics, construction, rubber, and paper printing [2]. Azo dyes are artificial colours that are often used as starting points in the textile production sector. Due to their availability, affordability, ease of synthesis, stability, and variety of colours, especially when compared to natural dyes, they are growing in popularity in the industrial sector [3]. Azo dyes make up the majority of synthetic dyes released into the soil and water. However, it has been noted that azo dyes are poisonous, resistant to heat and light, remarkably stable in acidic and alkaline conditions, and not biodegradable [4]. They additionally possess a capacity for persistence and accumulation at elevated quantities in the environment [5]. Due to the toxicity, carcinogenicity, and mutagenicity of these dyes as well as the constantly rising legislative limitations limiting effluent discharges, synthetic dye contamination of water bodies is a hazard for both the environment and public health [6]. When azo dyes are removed from textile production effluent using the current methods, which mostly use physicochemical methods, colour is eliminated [7]. These methods not only include the use of chemicals in the treatment process but are also costly, result in heavy sludge, and raise pollution issues in the future [7]. These drawbacks have motivated academics to hunt for novel, creative ways to remove colour from dye effluent. Biological techniques are seen as promising solutions because of their affordability, ease of use, amazing degrading efficacy, and ecological compatibility [5, 8]. Because they are more feasible, environmentally acceptable, and produce less hazardous metabolites, bio-friendly procedures have been given priority over physical and chemical ones in the remediation of dye-contaminated wastewater [8]. Numerous microorganisms from various taxonomic groups of bacteria, yeast, and fungi have exhibited the ability to decolorize colours by bioadsorption, biotransformation, or degradation [5]. A variety of bacteria have been studied for azo dye breakdown because of their rapid growth and degradation rates, considering that it has been shown that many of them produce colourless aromatic amines that are carcinogenic and mutagenic [5]. Certain bacterial cultures, on the other hand, can reduce azo compounds aerobically with the help of oxygen catalysed azo reductase, while also producing aromatic amines. Microorganisms can remove the dyes by employing various enzymes [6]. Because fungal enzymes are broad to specific dye structures, they can oxidise a wide range of colours [9]. Bacterial oxidoreductive enzyme systems such as azo reductase, DCIP-reductase, and laccase contribute to the biodegradation process both within and outside of the cells. It has been demonstrated that a number of bacteria can break down azo dyes in anaerobic environments. The decolorization of the precursors and their breakdown into colourless aromatic amines are the results of many of these bacteria, which have been extensively studied and proven to be extremely effective in the initial treatment of many azo dyes [10]. The elimination of these aromatic compounds produced as metabolites during biodegradation is required for the entire procedure to be complete. This is important because these biotransformation products have been demonstrated to be toxic, as well as carcinogenic and mutagenic in some situations [10]. The current work sought to isolate a *P. aeruginosa* strain that has capacity to detoxify the toxic dye. The potential of the isolate was evaluated to decolorize methyl red. For better methyl red decomposition, the consequences of various physical and chemical variables were also examined. Bioremediation efficiency of the isolate was also analysed using alginate gel beads followed by *Allium cepa* test.

## Materials and methods:

## Materials:

High-grade and high-quality chemical reagents included nutritional broth, glucose, ethyl acetate, hydrochloric acid, sodium hydroxide, n-hexane, sodium chloride, cobalt chloride, magnesium chloride, zinc chloride, mercuric chloride, Alginate, and others. The additional chemical reagents were bought from the German company Sigma Aldrich.

## Preparation of dye solution:

A 500 ml of distilled water and 0.10 gm of methyl red dye were combined to create the stock solution. Stock solution was used to create dye solutions with the desired concentrations (10-80 ppm). As azo dyes are unstable to moist-heat sterilization, the dyestuff solution was filtered using a 0.22 µm membrane filter.

## Medium and culture condition:

The basic medium used was mineral salt medium (MSM), which included 2 g (NH4)2SO4; 4 g K2HPO4; 0.5 g MgSO4 7H2O; 4 g KH2PO4; 0.01 g FeSO4 7H2O, and 0.01 g CaCl2; per litre of distilled water. Most of the experiments were carried out in 250 ml Erlenmeyer flasks containing 100 ml MSM with 0.1% glucose, 0.4% yeast extract, and 50 mg/ml dye.

## Bacterial strains isolation:

Samples of soil were collected from waste dump sites from Ha'il region, Saudi Arabia. Different batches of soil samples were used in this study. Initially, 5 grams of the soil sample were mixed with 100 milliliters of distilled water and left to settle after being vigorously agitated. The enrichment method was then used to isolate the desired bacterial strain, following which colonies were observed on Pseudomonas agar containing toxic dye methyl red, and their ability to grow on increasing levels of toxic dye (10 - 80 ppm) was checked. Eventually, a single clone *Pseudomonas aeruginosa* AM-1 that displayed the best growth was chosen for further analysis.

## Effect of methyl red dye concentration on biodegradation:

Isolated *Pseudomonas aeruginosa* strain AM-1 was cultured for 24 hours in different test tubes (8) with 10 ml of nutritious broth to examine the effect of dye concentration on breakdown. In each test tube 5 ml of solution dye was transferred to each test tube after the culture had grown with concentrations ranging from 10 - 80 ppm. Solutions (controls) made by 10 ml of nutrition broth also includes 5 ml of dye were prepared for each concentration. After three days, the culture mixture comprising the products of dye degradation was centrifuged at room temperature for 10 minutes at 10,000 rpm. The material was filtered via filter paper with a pore size of 0.2 µm. The extracted supernatant mixture's final absorbance value was determined to be 430 nm using a UV-visible spectrophotometer. After 6 days of incubation, the decolorization percentage (%) for each dye was measured at maximum max (430 nm).

Decolorization /Degradation (%) = [(Ao - Af)/Ao] x 100 (1) where Ao is initial absorbance and Af is final absorbance.

## Dye biodegradation based on temperature:

In order to ascertain whether temperature had an impact on the breakdown of methyl red, 10 ml of solution was given to eight test tubes, and all tubes were subsequently infested with the selected culture. Each test tube also received five mL of the 40 ppm methyl red stock solution after the proliferation of *P. aeruginosa* strain AM-1. Using 10 ml of nourishing broth and 5 ml of methyl red, a control solution was also created. Test tubes were incubated in the incubator at 18 - 53°C. The degraded samples were put into the test tubes and centrifuged for 10 minutes at 10,000 rpm and pass through with filter (0.2 µm). The following stage involved filtering each sample.

## Glucose effect on dye biodegradation:

For bacteria, glucose serves as both a major source of energy and a carbon source (a source of carbon atoms). After 1 day incubation, 5 mL of dye solution and various concentrations of glucose (100-1400 mg/l) were introduced to each inoculated test tube containing the dye solution. *Pseudomonas aeruginosa* strain AM-1 was cultured in nutrient broth media in test tubes and incubated at 37°C. For all concentrations of glucose, control solutions consisting of 10 mL of media and 5 mL of dye solution were also made. Followed by centrifugation at 10000 rpm for 10 mins and filtration through 0.2 µm filter, the deteriorated sample was tested using UV-visible spectrophotometry. Using the same procedure as in Equation (1), the proportion of the supernatant that had changed color was assessed.

## Effect of pH on dye biodegradation:

A constant concentration of methyl red (50 ppm) in 50 ml of MSM supplemented with 0.1% glucose and 0.4% yeast extract was tested under static conditions and at 30°C for 24 hours to determine the influence of different pH values on the decolorization. An optimum pH is required for bacterial growth. Thus, nutrient broth (sterile) was added in 14 test tubes and *P. aeruginosa* AM-1 strain was inoculated and kept incubator at 37°C overnight. Then 5 mL dye solution from stock solution (50 ppm) was introduced to all the test tubes. Test tube without dye serves as control. The pH in control test tube and test samples were adjusted by 1 M NaOH and 1 M HCl solution. The pH values were checked by pH indicator strips. Then each sample was centrifuged the pass through 0.2 µm filter. After 3 days the percent decolorization decreased in supernatant was analyzed by the above-mentioned method using a UV-Visible spectrophotometer.

## Temperature impact on dye biodegradation:

Effect of temperature on the degradation of methyl red dye was analyzed after overnight incubation and the bacterial growth is monitored. The 10 ml of bacterial culture was transferred to test tube and 5 ml methyl red dye solution (50 ppm) was transferred to each test tube. Control solution having 5 ml methyl red dye and 10 ml nutrient broth was also prepared. Test tubes were incubated at 18, 23, 28, 33, 38, 43, 48, and 53°C in the incubator. Wait for 72 hrs incubation samples were centrifuge for 10000 rpm x 10 mins followed by the filtration (0.2 µm). Percent decolorization was estimated as mentioned above.

## Sodium chloride effect on dye biodegradation:

Sodium chloride salt has an impact on the activity of dye degradation because it is a substantial salt that makes sea water more salinous. In ideal saline conditions, contaminants and dyes typically degrade. Test tubes containing the *P. aeruginosa* AM-1 culture were filled with the dye solution (5 ml). Each inoculated tube also received additions of sodium chloride in various concentrations (100-1400 mg/l). For each sodium chloride concentration, reference solutions were also made. The amount of decolorization was calculated using the supernatant that was collected after centrifugation.

## Impact of incubation time on dye biodegradation:

A 30 ml portion of nutrient broth was added to a large test tube, where it was then injected with a bacterial (*P. aeruginosa* strain AM-1) culture and left to incubate for 24 hours. After the culture had grown, 15 ml of dye solution were added. From one-day to fifteen days, the percentage of dye degradation was tracked. As a reference, a control solution comprising media (10 ml) and dye (5 ml) was also created. Until 15 days, the UV-visible spectrophotometer was used to measure the percent degradation rate.

## Effect of heavy metal ions:

Experiments were conducted with these metals present at concentrations of 1 and 5 mM to examine their effects on decolorization activity of *P. aeruginosa* AM-1. These metal ions included Co (CoCl2), Mg (MgCl2), Mn (MnCl2), Zn (ZnSO4), and Hg (HgCl2). Flasks containing the dye were then added after the cell suspension had been incubated for 15 minutes with ions of metal from stock solutions. At 30°C, decolorization was observed over a range of time periods.

## Bioremediation of methyl red dye:

The method of Khan and Ahmad [11] was used to trap the bacterial cells. Alginate (Manugel DJX), which was acquired from alginate industries in Hamburg, FRG, was used. To carry out the entrapment, 0.1 mL of cell (*Pseudomonas aeruginosa* strain AM-1) suspension was mixed with 0.9 mL of a 2% sodium alginate solution at room temperature, and the mixture was dripped from a syringe into 250 mL of 0.8 M calcium chloride solution. The resulting beads were taken out, washed, and then exposed to varying concentrations of toxic substances overnight at 37°C. Finally, the beads were removed from the toxicant solutions and examined to determine the degree of bioremediation.

## Toxicity evaluation of dye and its metabolites:

A. cepa test was used to analyse the detoxification of methyl red as described earlier with slight modifications [11]. To encourage the growth of roots, small, uniformly shaped A. cepa bulbs of equal size were originally subjected different methyl red dye (10 - 80 ppm) solutions that were obtained before and after passing through the immobilized AM-1 cell system, while water from aqua guard was used as a control. In each case, the bulbs were subjected to the appropriate treatment for 72 hours and the average length of 5 roots used as the measurement. Decrease in length of root of Allium cepa roots was considered as an indicator of toxicity.

## Statistical analyses:

Minitab software (Version 17) was used for statistical analysis and the graphical presentation of data for multifactorial designs.

## Results:

## Effect of dye concentrations on bacterial decolorization: UV–visible spectral investigation:

**Figure 1:**
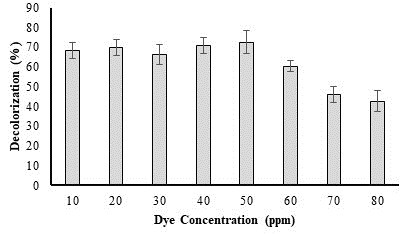
Effect of dye concentration on dye decolorization by *P. aeruginosa*.

Using UV-visible spectroscopy, decolorization analysis can determine whether dyes have been degraded. The absorption peaks obtained, and dye degradation are closely related. If the dye's primary UV-vis absorption peak vanishes or other new absorption peaks arise, this may indicate dye degradation. According to this study, *P. aeruginosa* AM-1 ability to remove colours diminished when the dye concentration rose from 10 - 50 ppm. Highest decolorization was achieved at 50 ppm dye concentration. It was discovered that, as (Figure 1) illustrates, the decolorization potential of *P. aeruginosa* AM-1decreased as dye concentration increased. As per research conducted by [12], *P. aeruginosa* AM-1 strain has a high potential to degrade azo dyes at initial concentrations. The high dye concentration, which led to a low bacterial growth rate, however, diminished that potential.

## Effect of pH on biodegradation of methyl red dye:

**Figure 2:**
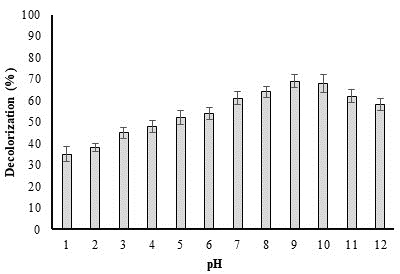
Effect of pH on decolorization of methyl red dye by *P. aeruginosa*.

**Figure 3:**
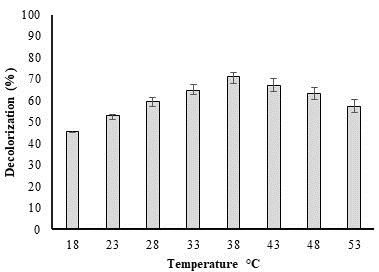
Effect of temperature on decolorization of methyl red dye by *P. aeruginosa*.

One of the most crucial factors determining bacterial degradation potential and enzyme activity is pH. Figure 3 illustrates how pH affects the decomposition of methyl red. When the pH moved from the acidic range towards the alkaline zone, there was an increase in deterioration. The rate of decolorization rose (62.32%) at pH 9 and it reduced at lesser pH, showing that severe alkaline as well as acidic environments had an impact on bacterial growth and enzymatic activity. Most often, alkaline environments with pH values between 6 and 10 are used to decolorize dyes (Figure 2)[13].

## Temperature effect on biodegradation of dye:

*P. aeruginosa* potential for methyl red dye (50 ppm) was influenced by temperature. (Figure 3) illustrates how temperature affects dye deterioration. Bacterial biodegradation potential diminishes as a result of the effect of temperature on bacterial growth. The largest decolorization (71.39%) was seen at 38°C, indicating that at temperatures that are higher or lower than this point, *P. aeruginosa* potential for decolorization reduces because of the slow development of culture. According to Anjaneya *et al.* (2011) [14], at high temperatures, bacterial enzymes become inactive, which significantly reduces the pace at which bacteria decolorize. According to Pearce *et al.* (2003) [15], the ideal growing temperature for decolorization of dye lies between 35 - 45°C.

## Influence of glucose concentration on biodegradation of dye:

**Figure 4:**
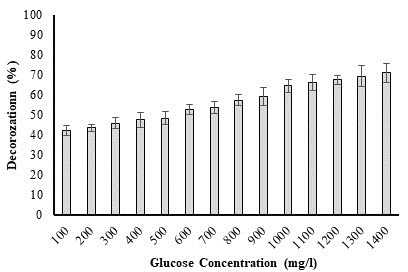
Effect of glucose concentration on percent decolorization of methyl red dye by *P. aeruginosa*.

Glucose is required by bacteria since it serves as both a carbon source and their primary source of energy. In context with the degradation capacity of bacteria, certain dyes are complex by nature and challenging to break down. As a result, additional glucose needs to come from a different source [16]. A rise in glucose concentration shows a beneficial impact on the decolorization rate. However, after a certain level, degradation activity decreases, probably due to a reduction in the capacity of the bacterial metabolic pathway to catabolize sugar [17]. Influence of glucose on degradation of methyl red is presented in Figure 4. A high degradation rate (71.08%) was seen after adding 1400 mg/l glucose in the present study.

## Effect of urea concentration on biodegradation of dye:

**Figure 5:**
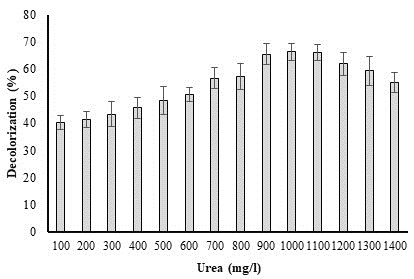
Effect of urea concentration on percent decolorization of methyl red dye by *P. aeruginosa*.

Bacteria require a lot of urea to break down the dye because they use it as a source of nitrogen. The effect of the urea concentration on *P. aeruginosa* capability for methyl red degradation is shown in Figure 5. At 100 mg/l, the chosen dye showed a significant rate of degradation (66.27%). Due to induced toxicity, the breakdown activity decreased as urea concentration rose. The percentage breakdown activity decreased to 59.59 percent at further higher concentrations as a result of urea toxicity and the rising urea level (1400 mg/l).

## Impact of incubation duration on dye biodegradation:

**Figure 6:**
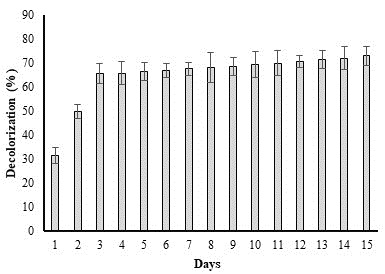
Incubation time (days) impact on percent degradation of methyl red dye by *P. aeruginosa*.

The degrading potential of bacteria is also impacted by time. The effects of time on *P. aeruginosa* breakdown of methyl red (50 ppm) are shown in Figure 6. The deterioration of the dye was observed every day for the first six days. Due to a lack of a discernible increase after 6 days, the percent degradation was measured every 3 days for a maximum of 21 days. A maximum in decolorization was seen after 3 days of incubation. After these 3 days, no appreciable decline was seen. Having entered the stationery and death phase is probably to blame for this.

## Effect of Salinity:

**Figure 7:**
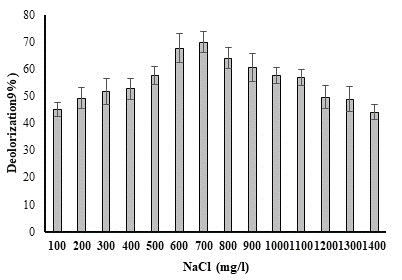
Effect of NaCl concentration on percent decolorization of methyl red dye by *P. aeruginosa*.

The effects of sodium chloride concentration on the degradation of methyl red (50 ppm) by a chosen bacterial strain (Figure 7). It is discovered that capacity of bacteria to break down dye declines as concentration rises. When *P. aeruginosa* degraded methyl red, the maximum percentage of degradation (69.87%) was discovered at a salt content of 700 mg/l. Bacterial cells undergo plasmolysis at high salt concentrations, which restricts bacterial development and, in turn, their capacity to degrade substances [16].

## Effect of heavy metal ions:

**Figure 8:**
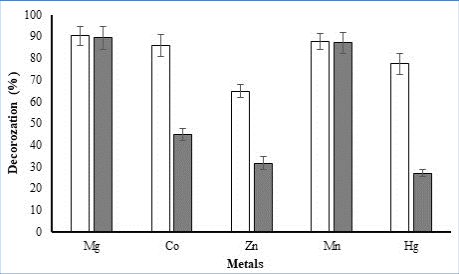
Effect of metal ions on decolorization of methyl red dye by *P. aeruginosa*. White and grey colour bars represent 1 mM and 5 mM concentrations of the metals, respectively.

Heavy metals are frequently present in textile effluents, making them more hazardous. The proteins or enzymes are directly affected by heavy metals because they interact with protein molecules to form produce complexes [18]. Therefore, the presence of two different concentrations (1 mM and 5 mM) of metals (Mg, Co, Zn, Mn, and Hg) were analysed for decolorization efficiency of methyl red (50 ppm) by *P. aeruginosa* Isolate. Metals Mg and Mn demonstrated a minimally inducing influence on decolorization performance (≥87%), as can be seen in Figure 8. Of the studied metal, Zn was the most inhibiting at both concentrations. Co and Hg both showed a considerable negative effect at 5 mM values.

## Bioremediation of methyl red dye:

The *Allium cepa* test was conducted using methyl red dye with varying concentrations. Alginate beads were exposed to varying concentrations of methyl red dye overnight at room temperature. Then beads were removed from the toxicant solutions and this solution was used to treat examined A. cepa L and determine the root length. The highest percent (91.1%) of bioremediation was achieved at 40 ppm dye concentration. The percent bioremediations were remarkably decreased from ≥60 ppm concentrations. This result exhibited that the *P. aeruginosa* strain AM-1 has capacity for bioremediation of methyl bye.(Table 1)

## Discussion:

Before it could be safely released into the environment, treatment of dyeing wastewater was highly important. Even more dangerous and damaging effects on the environment are being caused by these colour contaminants. Azolo dyes disrupt and reduce oxygen solubility and light penetration in water, which has an impact on plants' ability to photosynthesize. These dyes can have an aesthetic impact on the water's quality. These colours are harmful because of their chemical structure, which is naturally lasting and promotes a range of harmful effects.

Furthermore, these colours might lower rates of germination and might stop root expansion and seedling shoot. Additionally, these colours interfere with aquatic ecosystems' structure and threaten aquatic life's survival. Due to the potential for blocking sunlight from penetrating the receiving water, dye discharge without proper treatment may have an impact on aquatic life [20]. Hence, before being discharged into the environment, effluents and wastewater containing dye from the textile as well as related sectors should be cleaned. Biological enzyme systems, which are considerably less expensive and may be used under natural settings, have proven to be particularly effective in the breakdown and decolorization of dyes [21].

Textile dye biodegradation aims to remove colour, but it also transforms hazardous textile dyes into less toxic forms that can be released into the environment safely. The capacity of various bacterial strains to decolorize the dyes has been documented. Because each dye has a particular structure and level of complexity, the percentage of decolorization of various colours by bacterial strains varies [22]. Microbial cells have been observed to be hazardous to azo dyes at higher doses. As a result of the findings, higher dye concentrations may inhibit biomass growth via a variety of mechanisms, including microbial growth retardation via direct attack on enzymatic systems and adsorption of dye components on microbial cells. All of these mechanisms may affect metabolic pathways and cause shock loading rates of toxic secondary metabolites in the first few hours of the reaction [5].

High NaCl concentrations might promote biomass lyses, which would then provide enough organic carbon for efficient denitrification. Temperature, pH, dye concentration, and the availability of carbon are all physicochemical characteristics that influence the isolate's decolorization of textile colours [16]. In the current work, the ideal pH and temperature needed for *P. aeruginosa* to successfully decolorize the dye methyl red in liquid culture had been 9 and 38°C, respectively. These findings could be explained by the relationship between temperature and a rise in enzyme activity and growth in *P. aeruginosa* [16]. The isolate's ability to develop and subsequently decolorize methyl red was, however, shown to be quite constrained by additional temperature rise. The proportion of decolorization decreased as dye concentration increased more than 60 ppm may be due to increased toxicity which may leads to inhibit metabolic processes [22]. Utilizing the A. cepa test in which root growth was estimated provides the bioremediation capability of *P. aeruginosa* MW2 strain as suggest by the previous studies for other Pseudomonas species [11]. The toxicity assay also suggests that AM-1 strain *Pseudomonas aeruginosa* did not cause any harmful effect for the growth of A. cepa root growth and found to be environmentally friendly.

A broad spectrum of different physical and chemical methods of treatment is employed to process and remove dye from wastewater and effluents. Due to these difficulties, biological procedures that make use of green chemistry are viewed as practical alternatives for their efficiency in terms of cost, simplicity of utilization, and significant degrading efficiency [23]. The effectiveness of each parameter examined in this research was confirmed by researchers. The findings presented here call for additional research to determine the viability of these isolates for biodegradation and remedial applications, such as wastewater treatment. This bacterial strain has the potential to be used in the biological treatment of dyeing mill effluents due to its high degree of decolorization and ease of conditions.

## Conclusion:

The *P. aeruginosa* AM-1 strain demonstrated the ability to decolorize the methyl red dye color. Several toxicology studies indicate that the dye was cleaned up by bacteria after being processed, making it safe for the environment. Using *P. aeruginosa* strain AM-1 was optimized for pH, temperature, and salinity conditions, making it appropriate for use in practical situations. The ability of this strain to degrade various metals and bioremediation of methyl red dye is documented in this study. Further analysis in this direction using various toxic dyes and molecular level analysis would be helpful to obtain an efficient, cost-effective, environmentally accepted *P. aeruginosa* strains for the use in wastewater treatment.

## Figures and Tables

**Table 1:** : Estimation of percent bioremediation of methyl red by *P. aeruginosa* (AM-1)

**S.N.**	**Methylreddyeppm**	Rootlength*(cm)	**Bioremediation(%)**
1	Control	7.9±0.41	-
2	10	7.1±0.43	89.9
3	20	7.0±0.38	88.6
4	30	6.8±0.32	86.1
5	40	7.2±0.23	91.1
6	50	5.9±0.11	74.6
7	60	3.1±0.08	39.2
8	70	0.9±0.07	11.3
9	80	0.2±0.03	2.5
*Values are in mean ± SD.
